# Future prospects of deep learning in esophageal cancer diagnosis and clinical decision support (Review)

**DOI:** 10.3892/ol.2025.15039

**Published:** 2025-04-11

**Authors:** Aiting Lin, Lirong Song, Ying Wang, Kai Yan, Hua Tang

**Affiliations:** 1 School of Health Science and Engineering, University of Shanghai for Science and Technology, Shanghai 200093, P.R. China; 2 Department of Thoracic Surgery, The Second Affiliated Hospital of Naval Medical University, Shanghai 200003, P.R. China; 3 Department of Thoracic Surgery, Shanghai Pulmonary Hospital, School of Medicine, Tongji University, Shanghai 200433, P.R. China

**Keywords:** deep learning, esophageal cancer, precision medicine, early diagnosis, personalized treatment

## Abstract

Esophageal cancer (EC) is one of the leading causes of cancer-related mortality worldwide, still faces significant challenges in early diagnosis and prognosis. Early EC lesions often present subtle symptoms and current diagnostic methods are limited in accuracy due to tumor heterogeneity, lesion morphology and variable image quality. These limitations are particularly prominent in the early detection of precancerous lesions such as Barrett's esophagus. Traditional diagnostic approaches, such as endoscopic examination, pathological analysis and computed tomography, require improvements in diagnostic precision and staging accuracy. Deep learning (DL), a key branch of artificial intelligence, shows great promise in improving the detection of early EC lesions, distinguishing benign from malignant lesions and aiding cancer staging and prognosis. However, challenges remain, including image quality variability, insufficient data annotation and limited generalization. The present review summarized recent advances in the application of DL to medical images obtained through various imaging techniques for the diagnosis of EC at different stages. It assesses the role of DL in tumor pathology, prognosis prediction and clinical decision support, highlighting its advantages in EC diagnosis and prognosis evaluation. Finally, it provided an objective analysis of the challenges currently facing the field and prospects for future applications.

## 1. Introduction

Esophageal cancer (EC) represents a significant global health concern, frequently diagnosed at advanced stages due to its early symptoms being relatively concealed and easily overlooked by patients (1). This late diagnosis complicates treatment and contributes to poor prognosis, with numerous patients already in intermediate or advanced stages at the time of medical consultation. Regular physical examinations and targeted monitoring of high-risk individuals are crucial for improving early detection and accurate diagnosis, ultimately enhancing patient outcomes (2). EC is primarily classified into two types: Esophageal squamous cell carcinoma (ESCC) and esophageal adenocarcinoma (EA), which exhibit significant differences in etiology, incidence and treatment strategies. Continued research into biomarkers, therapeutic targets and screening strategies is essential for enhancing the management of EC and reducing its impact on global health (3). However, due to various limiting factors, such as inadequate early diagnostic technologies, limited treatment options and tumor heterogeneity, EC remains a complex and challenging disease (4,5).

Deep learning (DL) is an advanced machine learning (ML) technique characterized by a structure that typically consists of an input layer, multiple hidden layers and an output layer. The input layer receives raw data, such as medical images, while the hidden layers utilize complex neural networks for feature extraction and the output layer generates the final classification or regression results (6). DL offers advantages such as high accuracy, automatic feature learning, robust performance and real-time processing. By employing a multilayer architecture and nonlinear activation functions, DL models effectively capture complex data relationships and automatically learn representative features, thereby enhancing their generalization capabilities (7). In clinical settings, the enhanced capability for real-time analysis is especially crucial, enabling the effective identification and analysis of complex medical images and the capture of subtle pathological features. DL can flexibly handle diverse medical imaging data and integrate information from various sources, such as patients' historical medical records and other examination results, thereby assisting healthcare professionals in conducting comprehensive analyses and making personalized diagnostic decisions (8).

DL demonstrates a clear advantage in early EC diagnosis by exploiting extensive data training, especially when compared with novice endoscopists (9). DL models achieve multidimensional feature extraction through the analysis of large-scale medical imaging datasets (10). When processing video sequence data, these models demonstrate spatial-temporal analysis capabilities to capture subtle dynamic anomalies in mucosal microvascular morphology during respiratory and peristaltic movements. This overcomes the limitations of static, single-frame image analysis and reduces human-induced diagnostic discrepancies. It shows great promise in improving the accuracy and efficiency of EC detection (11). DL in medical image analysis has the potential to automate evaluation processes and provide quantitative imaging features associated with tumor heterogeneity (12). The present review summarized the relevant DL models for the diagnosis and prognosis of EC, aiming to explore the application potential of artificial intelligence in this field and its future development directions.

## 2. DL in revolutionizing endoscopic image screening and diagnosis

Upper gastrointestinal endoscopy plays an irreplaceable role in the monitoring and diagnosis of EC. Under endoscopic examination, the typical features of EC include mucosal ulceration, nodules, or stenosis, among others, and can be further classified based on the depth of invasion. Endoscopic examination and biopsy are the primary tools for identifying EC. Nevertheless, the diagnostic accuracy varies across different types of endoscopic techniques (13). White light imaging can provide high-resolution images, however, in certain cases, its ability to capture lesion details is insufficient and the image contrast is limited. To enhance the accuracy and efficiency of diagnosis, various new technologies such as narrow-band imaging (NBI) and blue-laser imaging (BLI) have been introduced into endoscopic practice, both of which can be paired with magnifying endoscopy (ME) and achieve a sensitivity of 95.2%. Notably, BLI offers slightly higher specificity (92.8%) compared with NBI (91.9%), with corresponding accuracies of 87.5 and 85.7%. In addition, optimizing endoscopic biopsy strategies markedly improves diagnostic efficacy: a single biopsy procedure achieves an accuracy of 93%, whereas seven consecutive biopsies can elevate accuracy to 98% (14). These technologies enhance the visualization of mucosal and vascular structures, when combined with DL algorithms, can assist doctors in detecting EC lesions (15).

Manual endoscopic diagnosis faces challenges such as accuracy, subjectivity, workload, and technology limits, hindering early disease detection and treatment. Integrating endoscopic images with DL offers a potential solution to these issues. Ohmori *et al* (16) developed a deep learning-based computer-aided diagnosis system for detecting and differentiating ESCC. The diagnosis of all lesions was confirmed through pathological evaluation of biopsy or surgical resection specimens. With non-magnifying endoscopy (non-ME) and NBI/BLI, its accuracy was 77%, slightly below the 78% of experts. With combined non-magnifying and magnifying endoscopy, its accuracy reached 83%, surpassing the 78% of experts (16). For different types of endoscopic images, Yang *et al* (17) developed two networks to examine ESCC: One for non-ME images based on YOLO V3 and another for ME images based on ResNet V2. The data included images captured from different perspectives and distances, as well as low-quality images with the presence of mucus or blood. The ME images were annotated based on the Japan Esophageal Society IPCL classification standard (AB classification) (18). This study demonstrated that the DL model was able correctly to diagnose the majority of cancer patients missed by endoscopists and improved the novice diagnostic average accuracy to 85.7% (17).

To improve the timely diagnosis of EC during endoscopic procedures, researchers are exploring real-time video analysis as a promising solution. With the single shot multibox detector (SSD) architecture, Shiroma *et al* (19) examined the ability of DL to detect T1 ESCC and all lesions in the images and videos during the training phase were confirmed based on pathological results. DL diagnostics were tested on two validation sets of 8,428 histologically confirmed EC images. The results showed that the system could detect superficial ESCC from EGD videos with high sensitivity. Furthermore, with real-time DL assistance, the sensitivity of endoscopists was markedly improved, showing an increase ranging from 5 to 25% (19). An encoder-decoder architecture-based SegNet model was employed to train on 6,473 NBI endoscopic images for pixel-level semantic segmentation. By generating probability heatmaps for dynamic lesion annotation, it offers a visual decision support tool for clinicians during endoscopic procedures. The study used pathological diagnostic results as the gold standard. In the static image dataset, the system demonstrated remarkable diagnostic performance with a sensitivity of 98.04% [95% confidence interval (CI): 96.87–99.21%], specificity of 95.03% (95% CI: 93.25–96.81%) and an area under the curve (AUC) of 0.989 (P<0.001). In video stream detection mode, the model maintained high single-frame sensitivity at 96.1% for magnified NBI images at ≥25 frames per second (fps). This technological advance overcomes the computational bottleneck in real-time high-definition video stream processing encountered by conventional CAD systems (20). Real-time video diagnosis still experiences limitations in stability and accuracy when processing high frame rate videos and motion blur (21).

Barrett's esophagus (BE) is a precursor to EA and traditional white-light endoscopy has a misdiagnosis rate of ≤23% in detecting EC, leading to delays in diagnosis (22). The endoscopic findings of BE include mucosal color changes, layered structures, glandular hyperplasia and surface irregularities (23). An artificial intelligence system based on convolutional neural networks (CNN) integrates i-scan imaging technology and combines pathological results with expert annotations to ensure diagnostic accuracy. This system achieves an accuracy rate of 86% in detecting and localizing early-stage tumors in BE, with an AUC of 93%, sensitivity of 91% and specificity of 79% on i-scan 1 images, markedly improving tumor recognition precision. Additionally, the system can analyze endoscopic images in real-time at 48 fps, with 97% of cases consistent with pathological and expert evaluations (24). In 2022, Knabe *et al* (25) employed artificial intelligence to assess the tumor staging of adenocarcinoma in BE using 1,020 images. The reference standard for the images was based on histopathology, with additional EUS evaluation when necessary. The results indicated that the accuracy of identifying tumor-free Barrett's mucosa was 85%, the accuracy of identifying mucosal carcinoma was 68% and the accuracy of identifying larger T3/T4 lesions was 73% (25). To evaluate the effectiveness of a DL system in detecting BE, Tsai *et al* (26) collected a large dataset of NBI images from two medical centers. EfficientNetV2B2 was selected as the core architecture, while resampling techniques were applied to increase the number of BE images, enhancing the model's ability to learn BE-specific features. The study used histological findings as the gold standard, comparing the endoscopic images with their corresponding biopsy or histological examination results to validate the accuracy of the model's predictions. The EfficientNetV2B2 system achieved an accuracy of 94.37% in detecting BE, with a sensitivity of 94.29% and a specificity of 94.44% (26).

In the process of disease diagnosis, determining the depth of tumor invasion is a critical step that influences the choice of treatment strategies and prognostic evaluation. However, in practice, the diverse appearance and growth patterns of EC, along with individual patient differences, markedly complicate the assessment (27). Consequently, an accurate determination often necessitates the integration of multiple diagnostic methods and professional expertise. DL particularly excels in the early detection of squamous cell carcinoma and adenocarcinoma, demonstrating capabilities comparable to endoscopists in identifying early-stage cancer and assessing tumor invasion depth (28). In 2019, Horie *et al* (29) developed a DL model for the diagnosis of early EC used CNNs. This model employed a deep neural network architecture with single detection multiple frames SSD, which is a DL model used for object detection. SSD achieves fast and efficient detection by simultaneously predicting multiple bounding boxes and class probabilities in a single forward pass. Using the histopathologic examination results of biopsies or resected specimens as the diagnostic gold standard and comparing them with the diagnostic accuracy of professional endoscopists (~89.7%), the overall diagnostic accuracy of the model for EC is ~93.1%, with a sensitivity of ~88.9% and a specificity of ~98.5% (29).

DL models also can analyze dynamic endoscopic video data in real time, learning to recognize relationships between tumors and surrounding tissues while automatically identifying and quantifying the depth of tumor invasion (15). Shimamoto *et al* (30) leveraged SSD as the mathematical algorithm and a 16-layer Visual Geometry Group (VGG) network as the foundation for their CNN-based DL model. The complexity of the image conditions, including variations in distance, angle and focus, is intentionally introduced to enhance the model's robustness. A unique training set of randomly extracted still images from 30 fps videos is used to assess ESCC invasion depth in real-time. All annotations and diagnoses adhere to the Japanese EC classification standards (31), systematically classifying pathologically confirmed cancers and subjecting them to meticulous manual annotation by experts. In both non-magnified endoscopic (NME) and ME images, artificial intelligence (AI) systems outperform experts in terms of accuracy and sensitivity. However, in specificity for ME images, the AI system achieves 95%, slightly lower than the 97% of the experts (30). This is the first instance of an DL system diagnosing EC invasion depth from video images, further expanding the scope of AI-assisted diagnosis in EC.

Accurate segmentation of lesions aids in assessing the size, shape and location of tumors, enabling physicians to identify cancer earlier and evaluate tumor characteristics. Li *et al* (32) proposed the You-Only-Have-One (YOHO) framework, focusing on the segmentation of early EC lesions. This method requires only a single patient image for training, thereby ensuring privacy and avoiding generalization issues. The results are determined using multiple image segmentation metrics, compared with physicians' manual annotations and blind evaluations from endoscopists. Through geometric data augmentation and an edge-enhanced UNet (eUNet), YOHO achieved an average Dice coefficient of 0.888, markedly higher than the 0.75 attained by traditional methods and demonstrated superior performance in metrics such as IoU, recall and precision. Image segmentation can be used to identify and locate lesion areas, blind evaluations indicated that its segmentation results received high recognition from clinical doctors (32). In 2023, Nakagawa *et al* (33) used deep neural networks to classify and determine the infiltration depth of ESCC. The system demonstrated a high accuracy (91.0%), with a sensitivity of 90.1% and a specificity of 95.8% in distinguishing between mucosal and microinvasion compared with deep submucosal invasion cancer. It is capable of completing the diagnosis of all validation images within 29 sec, compared with 70–180 min required for manual diagnosis (33).

Constrained by the limitations of storage capacity and power consumption on mobile devices, it is necessary to achieve model light weighting through parameter compression and computational optimization, all while balancing inference accuracy with operational efficiency. MobileNet V1 uses depth-wise separable convolutions to markedly reduce both the number of parameters and the computational cost (34). MobileNet V2 introduces an inverted residual structure and employs a linear bottleneck to further enhance the network's expressive capability (35). MobileNet V3 goes a step further by incorporating neural architecture search technology to optimize the network structure and by integrating an SE attention module (36). These series of optimizations make the model particularly outstanding for embedded applications such as image classification and object detection. Furthermore, the application of embedded system designs and modular deployment solutions can markedly reduce hardware dependency costs, facilitating flexible implementation of AI models across various clinical scenarios (Tables I and SI).

## 3. DL in computed tomography (CT) image screening and diagnosis unveiling hidden insights

In addition to endoscopic images, the use of CT images for EC detection has also demonstrated remarkable effectiveness. In early cancer screening, CT imaging has a diagnostic accuracy of 82.37%, which is lower than that of invasive endoscopic techniques such as colonoscopy or gastroscopy (14). The diagnostic performance of CT in EC staging revealed an accuracy of 66% (sensitivity, 61%; specificity, 68%) for T2 classification and 63% (sensitivity, 67%; specificity, 56%) for T3 classification. In detecting lymph node metastasis, CT demonstrated 65% accuracy, with sensitivity and specificity values of 59 and 75%, respectively (37). The distinction between CT and endoscopy lies in the ability of CT to more clearly depict the depth of tumor infiltration, the extent of the disease and the relationship between cancerous tissue and surrounding structures, as well as to identify distant anatomical features. Therefore, CT imaging plays a crucial role in the prognosis and treatment of EC (38).

Compared with traditional endoscopy and biopsy methods, non-invasive chest CT can be used for detection of EC at lower costs and with less trauma. Takeuchi *et al* (39) fine-tuned the VGG16 model to identify EC using CT images. The authors applied Gradient-weighted Class Activation Mapping to visualize the CNN's decision-making process, improving transparency and reliability. The experiment employed a double-blind cross-validation protocol, wherein two radiologists with over a decade of thoracic CT diagnostic experience (12 and 14 years respectively) independently performed image interpretations. In cases of diagnostic discrepancies, arbitration was conducted by a third-party senior expert possessing 20 years of clinical experience to ensure the precision of the results. The model achieved a diagnostic accuracy of 84.2%, with an F-value of 0.742, sensitivity of 71.7% and specificity of 90.0%. Compared with two radiologists, the AI system demonstrated higher accuracy and specificity, although its sensitivity was slightly lower (39).

By segmenting CT images, the anatomical structures and extent of lesions associated with EC can be clearly delineated, thereby enhancing the precision and efficiency of diagnosis. Non-contrast CT Images refer to images obtained during CT scans without the injection of any contrast agents. This approach can reduce patient risk and offers advantages such as rapid imaging and lower costs. These images primarily rely on the natural differences in tissue density to generate the images. For accurately segmenting, quantifying and detecting lesion thickness, Sui *et al* (40) employed a VB-Net architecture integrated into a CNN for processing non-contrast CT images, enabling automatic quantification of esophageal wall thickness and localization of lesion areas. The model performance was evaluated through rigorous partitioning of training and validation sets (7:3 ratio), long-term follow-up (two years) to confirm negative controls, pathological reconfirmation of missed diagnosis cases and an independent validation set with quantitative metrics including Receiver Operating Characteristic (ROC) curve (AUC=0.96), sensitivity (88.8%) and specificity (90.9%). With the assistance of the model, the diagnostic accuracy of radiologists increased from 53.6 to 75.8%. However, as the model primarily detects disease based on esophageal wall thickening, it still has limitations in identifying smaller lesions and indirect signs (40). Lin *et al* (41) proposed an automatic segmentation method for EC detection using non-contrast CT images, combining an improved no-new-Net model for three-dimensional esophageal segmentation with a decision tree classification model that incorporated esophageal wall thickness features. Independent validation demonstrated that the DL model attained an AUC of 0.890 (sensitivity, 90.0%; specificity, 88.0%), while the senior radiologist (Radiologist 3) showed significant AUC improvement to 0.965 (P=0.0068) with model assistance. Missed diagnosis analysis revealed that 70% were early-stage (T1-2) carcinomas, with all cases pathologically confirmed although no additional pathological review was conducted for missed cases (41). The study confirms that DL model can effectively assist radiologists in improving EC detection rates, demonstrating particular clinical value in reducing missed diagnoses during health screening examinations (42) (Tables II and SI).

## 4. DL applications in pathology transforming diagnostic landscapes

Pathological diagnosis primarily relies on microscopic examination of tissue samples, cellular samples, or biological fluids to identify the type of cancer, its degree of differentiation, metastasis and potential therapeutic targets, thereby assisting clinicians in determining cancer type, stage and prognosis. Traditional pathological diagnosis relies on manual observation and judgment, facing challenges in diagnostic accuracy and workload. By introducing DL models, researchers can achieve automated recognition and classification of complex tissue structures, thereby enhancing the ability for early EC detection (43).

Immunohistochemistry (IHC) is a pathological technique that uses specific antibodies to identify and localize antigens within tissues, revealing the distribution and expression levels of biomarkers. It holds significant importance for disease diagnosis, research and treatment planning. The expression of TFF3 can serve as a potential prognostic biomarker for cancer and is closely associated with tumor cell growth and migration. Gehrung *et al* (44) developed a semi-automated diagnostic system leveraging weakly supervised DL to enhance the early detection of BE. The study used endoscopic biopsy combined with the Prague criteria (≥C1/M3) as the gold standard for diagnostic outcomes, with low-quality or diagnostically uncertain samples (for instance, TFF3 staining ambiguity) subjected to secondary review by pathologists to ensure result reliability (45). This system processes Cytosponge-TFF3 test samples, achieving a 57% reduction in the workload of pathologists while maintaining high diagnostic accuracy, with diagnostic accuracy comparable with manual review (sensitivity, 81.7%; specificity, 92.7%). This method is not only applicable to Cytosponge-TFF3 testing but can also be extended to other pathological screening scenarios, such as fine-needle aspiration biopsies and bronchoalveolar lavage. The study provides valuable insights for the design of clinical decision support systems (44).

HER2 overexpression is typically linked to tumor aggressiveness, recurrence and poor prognosis. Pisula et al (46) introduced a DL-based scoring methodology designed to assess the overexpression of HER2 in gastrointestinal adenocarcinoma microscopic images. This approach incorporates an attention mechanism to discern vital tissue regions that markedly influence predictive outcomes. The HER2 status is determined by experienced pathologists analyzing the results of IHC HER2 staining, combined with fluorescence in situ hybridization testing for confirmation. The model achieved an accuracy of 0.94, a precision of 0.97 and a recall rate of 0.95 in the detection of HER2 status within EC tissues. Its efficacy was further substantiated through validation across datasets from various hospitals. In addition, by using morphological information from tissue images, the model demonstrated superior performance compared with traditional methods that rely exclusively on staining intensity (46).

Whole slide imaging (WSI) is a widely used technique in pathology that digitizes the entire surface of a tissue slide to create high-resolution digital images. This allows pathologists to view, analyze and diagnose tissue samples on a computer screen, eliminating the need for traditional microscopes. The integration of DL in WSI offers promising potential for enhancing diagnostic accuracy and efficiency in pathology (47). Faghani *et al* (48) employed an integrated image analysis approach, initially using the YOLO model to identify regions of interest (ROIs) within WSIs and concurrently conduct a preliminary classification of their corresponding histological types. Subsequently, these ROIs were further analyzed using a ResNet101 model pre-trained on the ImageNet dataset. In the test set, the sensitivity for low-grade dysplasia was 81.3% with a specificity of 100%, for non-dysplastic BE and with high-grade dysplasia, both sensitivity and specificity exceeded 90% (48). Similarly, Bouzid *et al* (49) demonstrated the effectiveness of a weakly supervised DL model for detecting BE from H&E-stained slides. Their model, trained and validated on samples from 1,866 patients across two clinical trials, can achieve diagnostic performance comparable with the TFF3 staining model without relying on localized expert annotations. The ROC AUC of the H&E model reached 91.4 and 87.3% in the discovery and external test datasets, respectively. This approach led to a 48% reduction in the workload of pathologists and a 37% decrease in TFF3 staining tasks, while preserving diagnostic performance (49).

EC metastasis refers to the process by which cancer cells spread from the primary tumor site to other parts of the body, typically occurring in regional lymph nodes (that is, lymph nodes near the esophagus) or distant organs such as the liver, lungs and bones. The presence of metastatic lesions is commonly confirmed through pathological examination. Wan and Zeng (50) constructed a predictive model for hepatic metastasis in EC based on ML. By analyzing clinical data from 17,800 EC patients in the SEER database, the authors identified 11 independent risk factors associated with hepatic metastasis and incorporated them into six ML classifiers to build the predictive model. The presence of metastatic tumors in the liver was confirmed through tissue biopsy and imaging examinations. Their GBM-based model showed excellent performance in internal validation, with AUC, accuracy, sensitivity and specificity of 0.885, 0.868, 0.667 and 0.888, respectively (50).

Lymph node metastasis (LNM) is one of the most common metastatic pathways in EC, particularly in advanced stages, where it is closely associated with prognosis. Chen *et al* (51) adopted a pre-trained ResNet50 model and transfer learning to extract deep radiomic features. The study involved a total of 308 patients, all of whom were pathologically confirmed cases of esophageal squamous cell carcinoma. These patients were divided into a training group (216 cases) and a testing group (92 cases) for retrospective analysis to evaluate the role of radiomic features in preoperatively predicting LNM in patients with ESCC. The AUC values of two radiologists, one with 15 years of experience and the other with 5 years of experience, were 0.67 and 0.61, respectively, which were lower than the AUC value of 0.80 of the optimal model (51). Squamous cell carcinoma in different organs may exhibit similar tissue characteristics when metastasizing to lymph nodes, allowing the model to demonstrate a degree of generalization for cross-organ detection of squamous cell carcinoma metastasis. Pan *et al* (52) developed a DeepLab v3 model using ResNet-50 as the backbone for automatic detection of LNM in squamous cell carcinoma H&E-stained slides. The LNM status is determined based on histopathological examination results, serving as the gold standard for evaluation. When tested on WSIs of esophageal lymph nodes, the model achieved a sensitivity of 99.2%, specificity of 93.0% and an overall accuracy of 94.0%. The model also demonstrated strong performance on untrained lung and laryngeal lymph node data (52).

Stanford University's MUSK model is pre-trained using the National Cancer Genome Atlas, which includes histopathological slides for 16 major types of cancer, along with related pathology reports and follow-up data. MUSK achieves a 75% accuracy in predicting disease-specific survival across all cancer types, compared with 64% with standard methods based on cancer stage and clinical risk factors. For melanoma recurrence prediction, MUSK has an AUC value of 0.87, an 8-point improvement over the best existing model. In pan-cancer prognosis prediction, its concordance index is 0.73, indicating strong clinical predictive capability (53). These results show that establishing a large, diverse and standardized pathological image dataset, supported by thorough clinical documentation, is essential for effective data analysis and model training. In practical applications, the auxiliary diagnostic functions of the AI system should be defined, workflows should be designed to encourage collaboration between pathologists and DL systems and smooth feedback channels should be established to make timely adjustments and optimizations to the system (Table III).

## 5. DL in therapeutic intervention pioneering novel treatment avenues

In the diagnosis and treatment of EC, DL technology assists clinicians in diagnosing different subtypes of EC. Additionally, DL plays a crucial role in pathological assessments by aiding in the evaluation of key characteristics, including tumor differentiation, depth of invasion and lymph node metastasis. Furthermore, DL can predict the responses of patients to neoadjuvant chemotherapy and radiotherapy by integrating image data with clinical information, thus facilitating the development of personalized treatment plans that improve therapeutic outcomes and optimize patient prognosis (54).

Neoadjuvant therapy is a treatment administered prior to the primary therapeutic intervention, aimed at shrinking tumors, lowering tumor staging and reducing the risk of recurrence, among other objectives (55). Given the individual variations in tumor morphology, size and location among patients, accurately predicting radiation dose distribution is essential for developing personalized treatment plans. This approach ensures that tumors receive the appropriate radiation dose while minimizing exposure to surrounding healthy tissues, thereby reducing the risk of side effects. Duan *et al* (56) developed and evaluated a novel three-dimensional DL model, AS-NeSt, which generates 3D dose distributions and dose-volume histograms for visualizing predictions. Using the clinical dose distribution as the ground truth for model predictions, the accuracy of AS-NeSt predictions was quantified through various dosimetric metrics [for example, the maximum dose, mean dose and dice similarity coefficient (DSC)]. The experimental team consisted of 1 junior dosimetrist with 1 year of experience and 1 senior dosimetrist with >5 years of experience. The application of the AS-NeSt model markedly reduces planning time and variability among dosimetrists, achieving a reduction of >61% in planning time for junior dosimetrists and 52% for senior dosimetrists. However, AS-NeSt's clinical application is constrained by its inability to incorporate the preferences of physicians, insufficient integration of patient background information and limited simultaneous integrated boost (SIB) case data.

To enhance clinical translation, it is essential to introduce personalized functionalities, integrate patient medical records and expand the SIB dataset to optimize model training. In addition, accurate delineation of the gross tumor volume (GTV) is crucial for dose distribution and target selection in radiotherapy. Automated delineation tools can assist physicians in more precisely defining tumor boundaries. Zhang *et al* (57) developed and validated a model based on the 3D version of nnU-Net for the automatic delineation of GTV in patients with ESCC. Here, two experts with >15 years of ESCC experience manually delineated GTV contours through consensus, serving as the gold standard to train and validate the AI model, ensuring clinical reliability. The model demonstrated strong performance in GTV delineation across three validation datasets, with median DSC values of 0.865, 0.876 and 0.866 and median average surface distance (ASD) values of 0.939, 0.789 and 0.875 mm, respectively. When assisting expert evaluations, 6 out of the specialists experienced an improvement in DSC values (P=0.003–0.048). Additionally, the model reduced the average manual delineation time for each tumor from 9.73 to 2.18 min, resulting in a time savings of 77.6% (57).

Following neoadjuvant chemotherapy, endoscopic evaluation of tumor response in EC patients primarily aims to assess the response of the tumor to chemotherapy and the treatment's effectiveness. The evaluation includes tumor size, morphology and the presence of residual tumor, which helps guide physicians in determining whether surgery is feasible or if further treatment adjustments are needed. Matsuda *et al* (58) adapted the AlexNet model for a binary classification task to evaluate the endoscopic response of EC patients after neoadjuvant chemotherapy. This adaptation aims to assess preoperative predictions regarding patient prognosis and the distribution of residual tumors post-surgery, thereby assisting physicians in making more precise treatment decisions. The experiment used the pathological evaluation results of surgically resected specimens as the gold standard for assessing endoscopic response. In the validation process, the AlexNet model achieved a specificity and positive predictive value of 100%, as well as an accuracy rate of 71%. In comparison, endoscopists demonstrated a sensitivity of 80%, specificity of 80% and an accuracy rate of 82.5% (58).

The tumor microenvironment (TME) is closely related to neoadjuvant chemotherapy, as various components within the TME, such as immune cells, blood vessels and extracellular matrix, directly influence the response of the tumor to chemotherapy. These characteristics can affect the efficacy of chemotherapy drugs. Li *et al* (59) not only addressed the heterogeneity of the tumor itself but also emphasized the influence of the TME on treatment response, offering a novel perspective for personalized treatment of EC. The authors employed four ensemble machine learning classifiers, incorporating three types of ROIs and a comprehensive group, to construct 16 distinct models. The study used preoperative CT radiomics to predict postoperative pathological complete response, with pathology as the accuracy standard. The training and testing datasets consisted of enhanced chest CT images of advanced EC, with a slice thickness of 5 mm, sourced from two medical centers. Among the 16 models, the ensemble XGBoost and ensemble random forest classifiers demonstrated the best performance, achieving average ROC AUC values of 0.906 and 0.918 on the training set, respectively. For the test set, the AUC values were 0.845 and 0.871, while for the external validation set, the AUC values were 0.650 and 0.749 (59).

To optimize personalized tumor therapy and fully exploit radiomics analysis for predicting patient responses to neoadjuvant chemotherapy and radiotherapy, it is often necessary to develop DL models that integrate the spatiotemporal information of tumors through the incorporation of time-series imaging data (60). This approach more effectively captures tumor dynamics and offers precise treatment recommendations at various stages (61). Furthermore, by iteratively refining the model based on feedback from therapeutic outcome assessments, the reliability of the model's predictions can be enhanced. In addition, integrating patient genetic profiles, clinical features and radiomic characteristics allows for a comprehensive consideration of individual patient variability, thereby enabling the design of more tailored treatment regimens (62) (Table IV).

## 6. Conclusion

The present review examined the application potential of DL in several critical areas of EC care. DL enhances the identification of early-stage EC and precancerous conditions in endoscopic image diagnosis. Using DL algorithms, DL improves the accuracy of image interpretation, leading to more reliable early detection. In CT image diagnosis, DL facilitates the differentiation between benign and malignant lesions with greater precision, which is essential for optimizing disease staging and informing treatment planning. DL plays a pivotal role in the analysis of tumor markers, which opens new avenues for non-invasive diagnostic approaches, thus allowing for the development of personalized treatment strategies tailored to individual patient profiles. These applications illustrate the transformative potential of DL in improving patient outcomes and highlight the need for its broader integration into clinical practice, offering clinicians vital tools for diagnosis, treatment and prognosis in esophageal carcinoma (15). DL has the potential to revolutionize the diagnosis and screening of EC, offering improved accuracy, efficiency and personalized care for patients (63).

DL integrates patient data to develop personal treatment strategies, optimizing therapeutic outcomes. Its efficient processing capabilities allow for rapid image analysis, thereby reducing workload and improving overall efficiency. Additionally, real-time monitoring provides immediate image analysis, ensuring timely recognition of changes in patient conditions. However, integrating DL into real-time endoscopy presents significant challenges. DL models can enhance the judgment of experienced endoscopists, but a notable gap remains between model development and clinical implementation. The future focus is on improving diagnostic accuracy through DL technology and big data analysis (64). The application of DL in EC research is expected to continue growing, with a need for further multicenter trials to assess its routine practice.

The application of DL technology in the management of EC faces multiple challenges, the most notable of which is its heavy reliance on data, as insufficient data can markedly impair the effectiveness of the model. To address issues such as image quality variability and the scarcity of annotated data, deep learning methods primarily employ data augmentation techniques such as rotation, flipping and scaling to enrich training datasets, enhancing the ability of the model to adapt to image variations under different imaging conditions. In addition, transfer learning reduces the dependency of new models on annotated data by leveraging pre-trained models. Multimodal learning, which integrates images from various imaging systems and uses the complementary information across different modalities, can markedly enhance the robustness of the model and improve its generalization capability in EC detection. However, these models still suffer from a lack of interpretability, which is further exacerbated by the complex hierarchical structures of DL models, ambiguous feature selection mechanisms and challenges in interdisciplinary communication. This opacity in decision-making processes makes it difficult for clinicians to assess whether the reasoning of the model is grounded in sound medical logic (65). Due to generalization issues, the adaptability across different hospital institutions is limited, raising concerns about external validity, while the presence of false positives and negatives still requires manual verification, further complicating clinical practice. Additionally, integration challenges related to technology and training hinder seamless incorporation into existing workflows.

To address these issues, recent research incorporating techniques such as local interpretable model-agnostic explanations and Shapley additive explanations, contextualized within clinical environments, offers methodologies that assist physicians in understanding the influence of specific features on model decisions (66,67). Furthermore, the deployment of visualization tools, such as heatmaps, to directly delineate the regions that the model focuses on, in conjunction with integrating causal analysis methods to enhance interpretability, further facilitates the comprehension of clinicians of the relationship between input features and the resultant outputs (68).

Physicians should be aware of the current clinical uses of DL in EC diagnosis and treatment to enhance patient care (69). Future research directions include the development of more robust algorithms and the enhancement of DL system interpretability, making them more transparent and reliable in clinical practice. Additionally, the integration of multimodal data, such as imaging, genomic information and clinical data, will facilitate a more comprehensive assessment of patient conditions, promoting the formulation of personalized treatment plans. The integration of multimodal data, leveraging its complementary properties, can enhance the effectiveness of clinical decision-making. The fusion of cross-modal features to build multidimensional evaluation models enhances the interpretability of treatment predictions. By incorporating clinical data characteristics with therapy response trajectories, dynamic predictive frameworks can be established to achieve more precise assessments of treatment efficacy and prognosis. Additionally, DL models, when processing high-dimensional and heterogeneous data, can uncover complex interrelationships and intrinsic patterns among the data, thereby providing a more scientific foundation for precision medicine.

## Supplementary Material

Supporting Data

## Figures and Tables

**Table I.** Summary of DL models in endoscopic image analysis for esophageal cancer screening.

							Accuracy, %		
								
First author/s, year	DL model	Image types	Train-set number	Tset-set number	Video	Form	Non-magnifying images	Magnifying images	(Refs.)
Horie *et al*, 2019	SSD	NBI and WLI	8,428	1,118	44	AI	93.10		(29)
Ohmori *et al*, 2019	CNN	NBI and BLI	22,562	727	-	AI + Experts	77.00	77.00	(16)
Yang *et al*, 2021	YOLO V3, Resnet	NBI and WLI/BLI	10,998	2,309	104	AI + Experts	99.50	88.10	(17)
Shimamoto *et al*, 2020)	SSD, VGG	NBI and WLI/BLI	23,977	23,977	-	AI + Experts	87.00	89.00	(30)
Shiroma *et al*, 2021	SSD	WLI/NBI	8,428		144	AI + Experts	85.00		(19)
Nakagawa *et al*, 2023	CNN	NBI/WLI	14,338	913	-	AI	91.00		(33)
Hussein *et al*, 2021	CNN	WLI	148,936	25,161	119	AI + Experts	86.00		(24)
Knabe *et al*, 2022	CNN	-	1,020	199	-	AI	85.00		(25)
Tsai *et al*, 2023	CNN	NBI	1,187	160	-	AI + Experts	94.37		(26)
Guo *et al*, 2020	SegNet	NBI	6,473	6,771	80	AI	-		(20)

DL, deep learning; SSD, single shot multibox detector; CNN, convolutional neural networks; VGG, visual geometry group; NBI, narrow-band imaging; WLI, white light imaging; AI, artificial intelligence.

**Table II.** Summary of DL models in CT image analysis for esophageal cancer screening.

First author/s, year	DL model	Train-set number	Tset-set number	Form	Research type	(Refs.)
Takeuchi *et al*, 2020	VGG16	1,457	146	AI + Experts	Single-center study	(39)
Sui *et al*, 2021	VB-Net	414	100	AI + Experts	Single-center study	(40)
Lin *et al*, 2024	CNN	645	200	AI + Experts	Double-center study	(41)

DL, deep learning; CT, computed tomography; CNN, convolutional neural networks.

**Table III.** Applications of DL in pathological analysis.

First author/s, year	DL model	Train-set number	Tset-set number	Form	Research type	(Refs.)
Gehrung *et al*, 2021	CNN	812	3,038	AI	Single-center study	(44)
Pisula *et al*, 2023	CNN	1,602	653	AI + Experts	Double-center study	(46)
Faghani *et al*, 2022	YOLO+ResNet101	368	70	AI + Experts	Single-center study	(48)
Bouzid *et al*, 2024	CNN	912	954	AI + Experts	Single-center study	(49)
Wan and Zeng 2024	CNN	12,460	5,340	AI + Experts	Single-center study	(50)
Chen *et al*, 2022	ResNet50	216	92	AI + Experts	Single-center study	(51)
Pan *et al*, 2020	ResNet50	242	855	AI + Experts	Single-center study	(52)

DL, deep learning; CNN, convolutional neural networks; AI, artificial intelligence.

**Table IV.** Research on DL in esophageal cancer therapy.

First author/s, year	DL model	Train-set number	Tset-set number	Form	Research type	(Refs.)
Duan *et al*, 2024	ResNet	236	160	AI + Experts	Double-center study	(56)
Zhang *et al*, 2024	nnU-Net	127	337	AI + Experts	Double-center study	(57)
Matsuda *et al*, 2020	AlexNet	193	20	AI + Experts	Single-center study	(58)
Li *et al*, 2024	CNN	201	30	AI	Double-center study	(59)

DL, deep learning; CNN, convolutional neural networks; AI, artificial intelligence.

## Data Availability

Not applicable.
