# Supplementary material for: Future prospects of deep learning in esophageal cancer diagnosis and clinical decision support (Review)
Source: Oncol Lett. 2025 Apr 11;29(6):293. doi: 10.3892/ol.2025.15039 (PMC12016012; doi:10.3892/ol.2025.15039)
Supplement: Supporting Data [file Supplementary_Data.pdf]

Table SI. Summary of applied references for Parts 2 and 3.

Author(s), year	Article title	Equipment manufacturers	Camera system model	Video processor model	Dataset type	Expert experience (number, n)	Dataset source	(Ref.)
Ohmori *et al*, 2019	Endoscopic detection and differentiation of esophageal lesions using a deep neural network	Olympus Co, Tokyo, Japan; Fujifilm Co, Tokyo, Japan	1. GIF-RQ260Z<br>2. GIF-FQ260Z<br>3. GIF-Q240Z<br>4. GIF-H290Z<br>5. GIF-HQ290<br>6. GIF-H260Z<br>7. GIF-XP290N<br>8. GIF-Q260J<br>9. GIF-H290<br>10. EG-L590ZW<br>11. EG-L600ZW<br>12. EG-L600ZW7	-	Training dataset:<br>Non-amplified endoscopic images (non-ME): 9,591 images from pathologically confirmed superficial ESCC and 1692 images from non-cancerous lesions or normal esophagus.<br>Enlargement endoscopic images (ME): 7,844 images from pathologically confirmed superficial ESCC and 3,435 images from non-cancerous lesions or normal esophagus.<br>Validation dataset:<br>Non-amplified WLI images: 255 images.<br>Non-amplified NBI/BLI images: 268 images.<br>Zoom-in NBI/BLI images: 204 images.<br>Validation images were obtained from 135 patients.	Certified experts of the Japanese Society of Digestive Endoscopy (n=2): with 8-24 years of experience as doctors and perform 2,500 to 20,000 endoscopies.	Osaka International Cancer Institute	(16)
Yang *et al*, 2021	Real-time artificial intelligence for endoscopic diagnosis of early esophageal squamous cell cancer (with video)	PENTAX Medical	1. EG29-i10<br>2. EG-2990Zi<br>The optical enhancement device EPK-i7000 was also used for image acquisition	-	Non-amplified endoscopic (non-ME) images: 10,574 (including 3,067 early ESCC, 3,310 AEC and 3,657 benign lesions).<br>Scope-optical enhanced (ME-OE) images: 414 (including 297 early ESCC, 76 AECs, and 41 benign lesions).	Experienced endoscopist: With >10 years of experience in EGD and using ME-OE endoscopy for over 3 years.<br>Novice endoscopist: 3 to 5 years of EGD experience and 2 to 3 months of ME-OE endoscopy training.	Qilu Hospital of Shandong University, collected from January 2012 to December 2019.	(17)
Shiroma *et al*, 2021	Ability of artificial intelligence to detect T1	Olympus Medical Systems, Co, Ltd	-	GIF-H290Z	Endoscopic EGD images from the Cancer Institute in Tokyo, Japan were used in the study period from February 2016 to	Eighteen endoscopists (n=7 certified, n=11 non-certified) participated in	Cancer Institute Hospital, Tokyo, Japan	(19)

	esophageal squamous cell carcinoma from endoscopic videos and the effects of real-time assistance				April 2017.<br>A total of 8428 training images of histologically confirmed esophageal cancer lesions including 397 ESCC lesions (332 superficial and 65 advanced carcinomas) were collected.<br>Of the training images, 6,026 were acquired by WLI and 2,402 from NBI.<br>Low quality images due to halo, blur, out-of-focus, inadequate mucus and air were excluded.	multicenter validation studies.		
Guo *et al*, 2020	Real-time automated diagnosis of precancerous lesions and early esophageal squamous cell carcinoma using a deep learning model (with videos)	Olympus Medical Systems	GIF-H260Z	EVIS LUCERA CV260 (SL)/CV290 (SL)	Training dataset:<br>6,473 NBI images (acquired in 2017)<br>Premalignant lesions / early ESCC: 191 patients (2770 images)<br>Non-cancerous lesions: 358 cases (3,703 images), including oesophageal varices, ectopic gastric mucosa, oesophagitis, etc.<br>Validation dataset:<br>Image Verification (2018):<br>Dataset A (malignant): 59 cases (1480 images)<br>Dataset B (non-malignant): 2004 cases (5,191 images)<br>Video verification (2018-2019 acquisition):<br>Dataset C (lesion videos): 27 non-amplified videos + 20 amplified videos<br>Dataset D (normal esophageal video): 33 cases (including 3 amplified videos)	Endoscopist (n=1): with at least 5 years of experience in EEC, was responsible for marking malignant image boundaries.<br>Senior endoscopists (n=3) participated in the validation of video frame-level sensitivity and specificity.	China: West China Hospital (mainly contributing to precancerous lesions, ESCC and some benign cases)<br>India: Jaswant Rai Specialized Hospital (providing images of normal esophagus)<br>USA: San Bernardino Gastroenterology Associates (provide image of normal esophagus)	(20)
Hussein *et al*, 2021	A new artificial intelligence system successfully	Pentax （Hoya, Japan）	1. OPTIVISTA plus<br>2. EPK-i7000<br>3. EG-2990i	-	Training set: 148,936 video frames (from 31 patients with dysplasia, 31 with NDBE patients, 2 normal oesophagus).	Endoscopy experts (n=6): >5 years of BE endoscopy experience with weekly	4 European expert centers (UK, Spain, Belgium, Austria)	(24)

	detects and localises early neoplasia in Barrett's esophagus by using convolutional neural networks				Validation set: 25,161 images (11 patient videos).<br>test set:<br>Classification model: 264 i-scan-1 images (28 dysplasia patients, 16 NDBE).<br>Segmentation model: 86 i-scan-1 images (28 patients with dysplasia).	endoscopic therapy, in accordance with ESGE guidelines.<br>Pathologist (n=1): A BE specialist pathologist with >10 years of experience, responsible for histological review (dysplasia cases was independently confirmed by two experts).<br>Non-expert control endoscopists (n=6): 3 years of experience, participated in the performance comparison		
Knabe *et al*, 2022	Artificial intelligence-assisted staging in Barrett's carcinoma	Fujifilm Corp., Tokyo, Japan	Fujifilm 600 and 700 series	-	A total of 1,020 images were used from 577 patients (at least one per patient, up to three).<br>The final images used for training were 821, and the validation set was 199.<br>Images are labeled according to the corresponding histopathological results, covering different cancer stages (e.g., T1a sm1, T 1 bT2, T3 T4, etc.)	-	1. Institute of Biostatistics and Mathematical Modeling, Goethe University of Frankfurt<br>2. Department of Gastroenterology, Sana Klinikum GmbH<br>3. HMS Analytical Software GmbH, Heidelberg, Germany<br>4. Department of Medicine I, Asklepios Paulinen Klinik Wiesbaden	(25)
Tsai *et al*, 2023	Artificial intelligence system for the	Olympus Medical Systems,	NBI mode	-	Dataset composition:<br>Training set: 771 images, 563 without BE and 208 with BE.	Supervising physicians of the Taiwan Society of Digestive	Chung Shan Medical University Hospital (located	(26)

	detection of Barrett's esophagus	Co, Ltd			Validation set: 193 images, including 141 without BE and 52 with BE.<br>Test set: 160 images, including 90 without BE and 70 with BE (confirmed by histology).<br>Due to the small number of BE images in the dataset, the data augmentation technique was used to increase the number of BE images in the training set from 208 to 624 to improve the training effect of the model.	Endoscopy (n=3), were responsible for the independent annotation and classification of the images.	in Taichung City, Taiwan)<br>Changhua Christian Hospital (located in Changhua County, Taiwan)	
Horie *et al*, 2019	Diagnostic outcomes of esophageal cancer by artificial intelligence using convolutional neural networks	Olympus Medical Systems, Co, Ltd	1. GIF-H290Z<br>2. GIF-H290<br>3. GIF-XP290N<br>4. GIF-H260Z<br>GIF-H260	1. EVIS LUCERA CV-260/CLV-260<br>EVIS LUCERA ELITE CV-290/CLV-290SL	A total of 8,428 training images of esophageal cancer lesions were collected from 384 patients. The images include 397 lesions of ESCC and 32 lesions of esophageal adenocarcinoma. Additionally, the test image set contains images of 49 esophageal cancer lesions from 47 patients.	Skilled physicians usually have more than 5 years of endoscopic operation experience and have performed a large number of endoscopies in high-flow cancer centers (e.g., >3,000 times)	Cancer Institute Hospital, Tokyo, Japan	(29)
Li et al, 2024	Single-image-based deep learning for segmentation of early esophageal cancer lesions	Fujifilm Corp., Tokyo, Japan; Olympus Medical Systems, Co, Ltd	GIF-H290Z<br>GF-UCT260<br>CV-1500<br>VP-7000<br>SU-9000H	-	The self-built data set EEC-2022: contains 1236 early esophageal cancer images, which were annotated by students under the supervision of experienced endoscopist and divided into 1092 training set and 138 test set.<br>Public polyp dataset CVC-ClinicDB (also known as CVC-612): contains 612 images, of which 550 were divided into training sets and 62 test sets according to PraNet standards.<br>Public polyp data set Kvasir-SEG: provides 1,000 images with different resolutions, which is also divided into 900	-	China: West China Hospital of Sichuan University	(32)

					training set and 100 test set according to the PraNet division strategy.			
Nakagawa *et al*, 2023	Classification for invasion depth of esophageal squamous cell carcinoma using a deep neural network compared with experienced endoscopists	Fujifilm Corp., Tokyo, Japan; Olympus Medical Systems, Co, Ltd	GIF-XP290N, GIF-Q260J, GIF-RQ260Z, GIF-FQ260Z, GIF-Q240Z, GIF-H290Z, GIF-H290, GIF-HQ290, GIF-H260Z, EG-L590ZW, EG-L600ZW, EG-L600ZW7	1. CV260<br>2. VIS LUCERA CV-260/CLV-260<br>3. EVIS LUCERA ELITE CV-290/CLV-290SL<br>4. LASEREO	training set:<br>Non-magnified images (non-ME): 8,660 images (white light, NBI, iodine staining)<br>Enged image (ME): 5,678 (NBI)<br>Pathological classification: EP / LPM, MM, SM1, and SM2 / 3 cancers<br>Time range: December 2005-December 2016<br>validation set:<br>Non-magnified images: 405 images<br>Magnified images: 509 images<br>Time range: January 2017-April 2018	Experts involved in the evaluation (n=16): Certified by the Japanese Society of Digestive Endoscopy.<br><br>Experience: 9-23 years<br><br>Operating volume: 3,000-20,000 endoscopies completed	Osaka International Cancer Institute	(33)
Takeuchi *et al*, 2020	Performance of a deep learning-based identification system for esophageal cancer from CT images	GE Medical Systems; Canon Medical Systems Corporation	1. Revolution CT<br>2. GE Discovery CT750 HD<br>3. 64-slice LightSpeed VCT<br>4. Aquilion 64<br>5. Aquilion one	-	Total training data: 1500 pieces (cancerous + normal), divided into training set and test set by 9:1.<br>Test set: 46 cancerous images + 100 normal images.	Senior radiologists (n=2): >10 years of experience in CT diagnosis.	457 patients with primary esophageal cancer admitted to Keio Ying University Hospital, Japan (2005-2018)	(39)
Sui *et al,* 2021	Detection of Incidental Esophageal Cancers on chest CT by deep learning	Toshiba Medical Systems; Siemens Healthcare; GE Healthcare	Parameters include:<br>Floor thickness: 5 mm<br>Image Matrix: 5,12,5,12<br>Tube voltage: 120 kVp	-	Data Set 1 (February 2017, April 2019):<br>Training set: 141 esophageal cancer + 273 negative (414 total)<br>Validation set: 7:3 ratio (unspecified value, estimated as ~ 99 cancer + 191 negative)<br>Data Set 2 (January 2017, December 2019):<br>52 missed esophageal cancer + 48 normal (100 in total)<br>Total cases: 514 (414 + 100)	Radiologists (n=3): 5-7 years of experience in CT diagnosis.	China-Japan Friendship Hospital of Jilin University, China.	(40)
Lin *et al*, 2024	Esophageal cancer detection via	GE Medical System;	1. Revolution CT<br>2. Discovery CT750	-	Training set: 397 patients with oesophageal cancer + 250 healthy	The radiologist team: Senior physician		(41)

non-contrast CT and deep learning	Canon Medical Systems Corporation; United Imaging	HD<br>3. LightSpeed VCT (512 layer)<br>4. Aquilian one<br>5. uCT 760 (128 layer)		individuals (647 patients in total)<br>Validation Set: 100 patients with oesophageal cancer + 100 healthy individuals (200 patients in total)	(n=1): 13 years of experience in imaging diagnosis;<br>Junior physicians (n=2): 5 years of experience in imaging diagnosis.

BE, Barrett's esophagus; EEC, early esophageal cancer; ESCC, esophageal squamous cell carcinoma; WLI, white light imaging; NBI, narrowband imaging; BLI, blue laser imaging; AEC, advanced esophageal carcinoma; EGD, esophagogastroduodenoscopy.
